# *Clinics and Practice*: A Rapidly Developing Journal

**DOI:** 10.3390/clinpract12060098

**Published:** 2022-11-18

**Authors:** Giustino Varrassi

**Affiliations:** Paolo Procacci Foundation, 00193 Rome, Italy; giuvarr@gmail.com

Recently, MDPI has acquired a new journal, *Clinics and Practice*, and started its development. The indicated aims of the journal are many, probably too many, because the title is extremely wide. In any case, it is especially devoted to the clinical aspects of medicine, not disregarding the basic and translational research.

Publications on clinical medicine, including pain medicine, are certainly the main scope of the journal. The interest is also on infectious diseases, oncology, sport medicine and reproductive medicine, including pediatrics. Moreover, it is open to dentistry, psychology and psychiatry, public health, and healthcare, and lastly, but not of a minor interest, to intensive care. Of course, publications on basic and clinical pharmacology represent another important aspect of this evolving journal. The readers may find more detailed information at the link: https://www.mdpi.com/journal/clinpract/about (accessed on 15 November 2022). 

The Editorial Management of the journal has kindly invited me as Editor in Chief. After an attentive analysis of contents, recent publications, and the website, and taking into consideration several other personal aspects, I decided to accept the challenging and intriguing adventure.

A new journal is always an intriguing adventure. In this case, the main attractive point was the great rapidity and proactivity of the publisher, and its expanded and growing offer of communication tools in science. The interactivity and potentiality have a clear example in what they call “Topics” (https://www.mdpi.com/topics?query=&journal=clinpract&status=all&category=all (accessed on 15 November 2022)). This is a part of the website whose aim is to invite anyone to a transversal interaction on a specific topic in the scientific discussion. Those interested may easily find a common arena to have an open and stimulating confrontation. This is an interesting potentiality, providing the possibility to compare our point of view with many others having our same objective: the growth of science via research and interaction with peers.

Going back to *Clinics and Practice*, and looking at its evolution in the last 2 years (Figure 1), it seems as though this journal is going to have a bright future. At the moment, it is already on PubMed and WoS. Presumably, it is going to have an impact factor in 2023, when it should also be on Scopus. The presence in important data banks is a first step for a healthy and progressive growth. 

The contents of the journal are definitely interesting. In the last two years, there have been important papers already cited several times. The most important between them is a narrative review on the interferences between COVID and hypoxia, with over 200 citations [1]. Then, there is a further publication addressing variants of COVID-19 [2]. COVID in the last years has attracted many scientists, as evident from many other published articles on different aspects and countries [3,4,5,6]. Of course, the journal has published several other articles of interest, and widely cited; e.g., there is an intriguing article, cited around 100 times, connecting the effects of microbiota with mental health [7]. This is a topic of great appeal, considering the always new data found in the literature on the importance of microbiota [8].

*Clinics and Practice* has a peculiar column “Editor’s Choice Articles” listing all the recent articles preferred by the editorial team. This is appealing, considering that also indicates how many times the article has been viewed on the website and cited. Between them, the best ranked have over 5000 views [1,2], but there are others with over 2000 views in short time [9,10]. Now, this column is just maintained by the editorial office, but it could be a fantastic arena to involve the readers and ask them to express their opinions and preferences.

A wise and professional management will certainly provide an organic and progressive evolution of the journal. It will be my pleasure to give most of my energies and enthusiasm to help in the process. I count on the support of all my colleagues, the members of the Editorial Board, to make sure that the quantitative and qualitative growth will continue and increase as much as possible. *Ad maiora*.

## Figures and Tables

**Figure 1:**
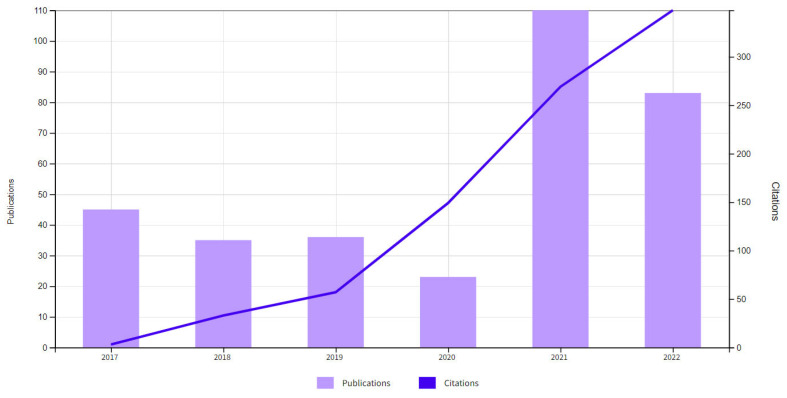
Web of Science (WoS) report on the evolution of *Clinics and Practice* in the last few years. The increase in number of published articles, but especially in the number of citations of the published articles is more than evident.
